# Diagnosis and treatment of brain injury complicated by hypernatremia

**DOI:** 10.3389/fneur.2022.1026540

**Published:** 2022-11-28

**Authors:** Hao Wu, Ming Bai, Xiayin Li, Yan Xing, Shiren Sun

**Affiliations:** ^1^Department for Postgraduate Students, Xi'an Medical University, Xi'an, China; ^2^Department of Nephrology, Xijing Hospital, Fourth Military Medical University, Xi'an, China

**Keywords:** hypernatremia, traumatic brain injury, conventional treatment, continuous renal replacement therapy, reduction rate of serum sodium

## Abstract

Hypernatremia is a common electrolyte disorder in patients with brain injury. The mortality of brain injury patients with severe hypernatremia may be as high as 86.8%. The efficacy of conventional treatment for hypernatremia is limited. Continuous renal replacement therapy (CRRT) can slowly, controllably, and continuously reduce the blood sodium concentration and gradually become an important treatment for severe hypernatremia patients. This review aims to provide important information for clinicians and clinical researchers by describing the etiology, diagnosis, hazards, conventional treatment, and CRRT treatment of hypernatremia in patients with traumatic brain injury.

## Introduction

Every year, more than 55 million people worldwide suffer from different degrees of brain injury, and brain injury not only leads to concussion, cerebral hematoma, brain hemorrhage, and other brain parenchymal injuries but is also often complicated with hypernatremia (1, 2). The prevalence of hypernatremia in patients with brain injury is more than 35% (3, 4). Hypernatremia affects the functions of the nervous, endocrine, circulatory, immune, and other systems and increases hospitalization costs, length of stay, and mortality (5).

The mechanism of hypernatremia in brain injury patients is complicated and includes central diabetes insipidus, osmotic therapy, iatrogenic sodium load, insufficient fluid intake, and excessive fluid loss (6). Treatment modalities must be selected according to different causes, subtypes, and severities of hypernatremia (7–10).

Continuous renal replacement therapy (CRRT) can effectively reduce blood sodium concentration and gradually become an important treatment for patients with severe hypernatremia (11). This review aims to provide important information for clinicians and clinical researchers by describing the etiology, diagnosis, hazards, conventional treatment, and CRRT treatment of hypernatremia in brain injury patients.

## Etiology and mechanism of hypernatremia in brain injury

### Central diabetes insipidus

**Figure 1:**
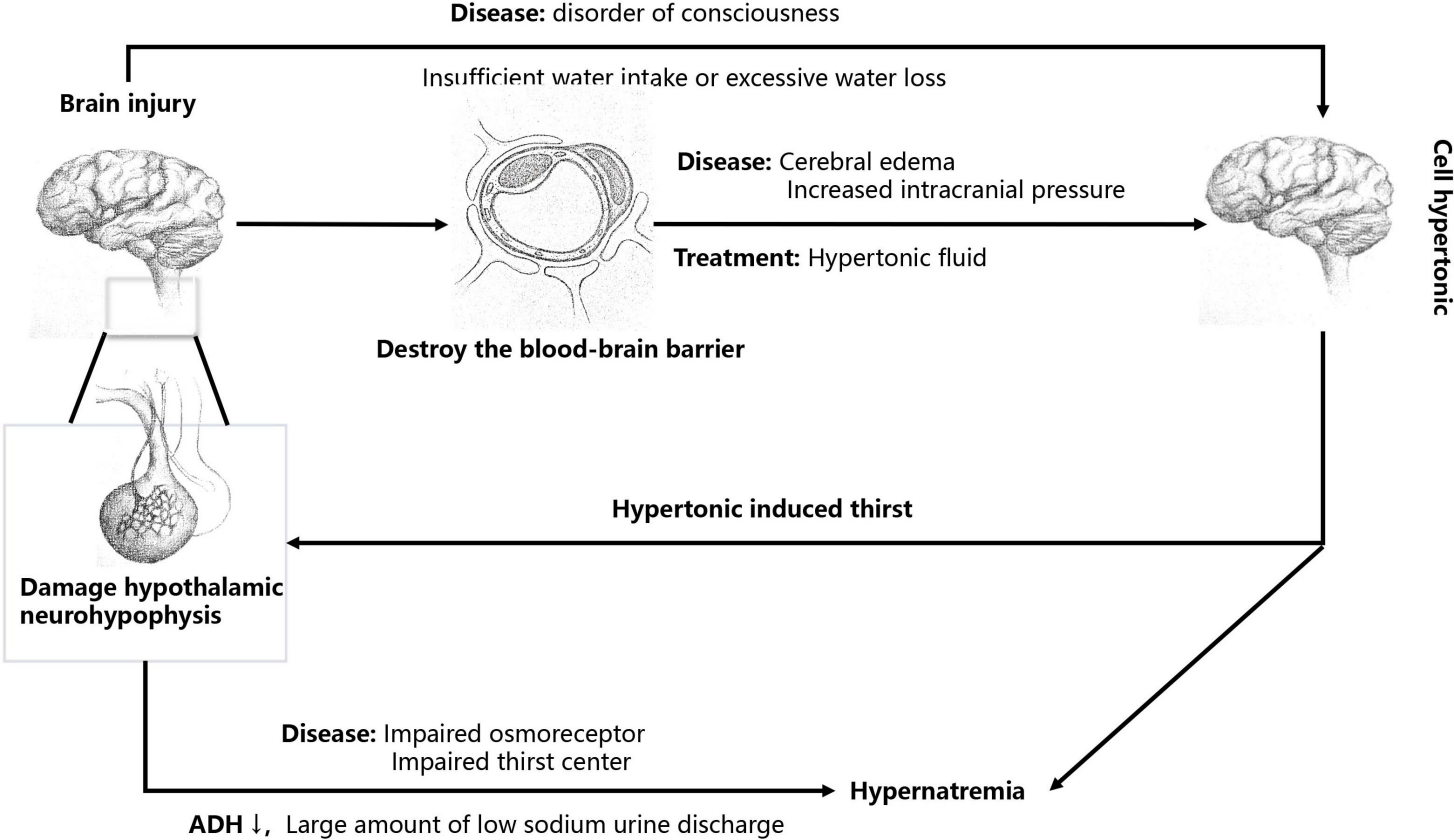
The mechanisms of brain injury in patients with hypernatremia.

Central diabetes insipidus is regarded as the main cause of hypernatremia in patients with brain injury (Figure 1). Brain injury involving the hypothalamus-pituitary gland may cause decreased secretion of antidiuretic hormone (ADH) and damage to the osmoreceptor or thirst center, resulting in a large amount of low-sodium urine discharge (central diabetes insipidus), causing hypernatremia. The increase in blood sodium caused by central diabetes insipidus is mostly normovolemic hypernatremia (12). If the thirst center was damaged, it would often cause moderate to severe hypernatremia, with a serum sodium concentration as high as 155–190 mEq/L (8, 13).

### Use of hypertonic liquid

Brain injury can disrupt the blood–brain barrier, leading to brain edema and increased intracranial pressure (Figure 1) (5). Osmotic therapy (mannitol or hypertonic saline) is initiated in patients with traumatic brain injury with cerebral edema and increased intracranial pressure after a series of treatments, such as cerebrospinal fluid drainage, have failed (14). The supplementation of hypertonic saline can increase the content of water and sodium, often leading to hypervolemic hypernatremia (5). Mannitol can also reduce intracranial pressure, but osmotic diuresis often leads to a disproportionate loss of sodium and water, resulting in hypovolemic hypernatremia (15).

### Iatrogenic sodium load

Patients with brain injury need sodium-containing drugs and liquids due to their illness, such as intravenous hypertonic saline, normal saline enema, high sodium enteral nutrition solution, and other liquids. The sodium load caused by these drugs can also lead to an increase in blood sodium, resulting in hypervolemic hypernatremia (7).

### Insufficient water intake or excessive water loss

Patients with brain injury often have an abnormal state of consciousness and are unable to drink actively, which can cause an increase in blood sodium if fluid supplementation is insufficient, often leading to hypovolemic hypernatremia. Alternatively, fever, mechanical ventilation, and tracheotomy can lead to increased water loss from the airways or skin, often leading to normovolemic hypernatremia if the lost water is not replaced in time (7, 16).

## Diagnosis and risks of hypernatremia in brain injury

At present, the commonly used diagnostic criterion for hypernatremia is serum sodium > 150 mEq/L (6, 15). In general, hypernatremia is defined as chronic when it persists for more than 48 h and acute when it lasts for < 48 h (17).

Acute hypernatremia has a more pronounced clinical presentation than chronic hypernatremia. Rapidly increasing sodium in the blood leads to the increased osmotic pressure of the extracellular fluid, which can result in intracellular water efflux and cell shrinkage, leading to a dramatic reduction in brain volume, causing focal intracranial hemorrhage and subarachnoid hemorrhage and irreversible neurological damage, thus causing disorders in the normal functioning of the central nervous system, and such patients may present with confusion, drowsiness, fatigue and dysphoria, convulsions, and coma (18). Patients with the more severe disease also experience osmotic demyelinating changes in brain tissue, similar to damage when chronic hyponatremia is corrected too quickly, and clinical symptoms usually appear 2–6 days after too rapid increases in serum sodium concentrations (19), manifested as extrapyramidal dysfunction, cognitive impairment, paraparesis or quadriparesis, seizures, mutism may occur if the disease progresses further, sensation and understanding are relatively intact, but they can only usually be expressed by turning the eyeball and blinking (18, 20). After hypernatremia enters a chronic process, with the transfer of water from the cerebrospinal fluid to brain tissue and the uptake of solutes by brain cells, water flows back into the cells, the brain adapts to the hypernatremia, and neurological symptoms may be relieved to some degree (8, 18).

The prognosis of patients with brain injury complicated by hypernatremia is closely related to the level of serum sodium, and the risk of death is positively correlated with the severity of hypernatremia (21). Hypernatremia is usually classified as mild hypernatremia (serum sodium 150–155 mEq/L), moderate hypernatremia (serum sodium 155–160 mEq/L), and severe hypernatremia (serum sodium 160 mEq/L). The more severe the hypernatremia was, the higher the risk of death (mild: HR = 3.4, *P* < 0.001; moderate: HR = 4.4, *P* < 0.001; severe: HR = 8.4, *P* < 0.001) (6). The mortality rates of brain injury complicated with mild, moderate, and severe hypernatremia were 20.6, 42.4, and 86.8%, respectively (15).

## Conventional treatment of hypernatremia in brain injury and reduction rate of serum sodium

### Conventional treatment

Identification of the cause of hypernatremia and assessment of the patient's volume status is the basis for treating hypernatremia. Hypovolemic hypernatremia is accompanied by loss of water and electrolytes. For patients with hemodynamic instability, an isotonic solution (0.9% NaCl solution) should be used for resuscitation until their vital signs are normal. After hemodynamic stability, the hypotonic solution is selected to correct the volume deficiency (7). Hypervolemic hypernatremia is usually associated with iatrogenic sodium load, and the first step is to stop the infusion of hypertonic drugs. Alternatively, using diuretics alone to excrete sodium will lead to more water loss, which will aggravate the hypertonic state. Therefore, using a 5% glucose solution (or sodium-free liquid) together with diuretics can reduce the capacity load and improve the hypertonic state simultaneously (Table 1) (8). Normovolemic hypernatremia is treated to correct dehydration because there is no concomitant loss of electrolytes (Table 1), which can be calculated by the following formula for water deficit, and then 5% glucose solution (or liquid without sodium) was supplemented to correct it; female water deficit = $0.5\times \text{body weight}\times \frac{actual\;sodium\;concentration-140}{140}$; male and child water deficit = $0.6\times \text{body weight }\times \;\frac{actual\;sodium\;concentration-140}{140}$ (7–9). If isovolemic hypernatremia is caused by central diabetes insipidus, desmopressin should be given to block the effect of ADH (Table 1). Desmopressin can be administered intranasally, orally, subcutaneously, or intravenously (22) and typically starts with an intranasal formulation. The initial dose for intranasal administration is 10 μg at bedtime, which can be adjusted in 10 μg increments depending on the condition, with a routine daily maintenance dose of 10–20 μg/day. The starting dose of the oral formulation is 50 μg at bedtime, with the total amount not exceeding 1,200 μg/day. The usual dose for the subcutaneous form is 1 μg/dose every 12 h; 1–2 μg desmopressin can also be intravenously injected within 2 min (22).

**Table 1:** Conventional treatment of brain injury with hypernatremia.

**Classification**	**Hypovolemic**	**Hypervolemic**	**Normovolemic**
Characteristics	Water loss > sodium loss (8, 9) (Use of diuretics or osmotic diuretic action)	Water, sodium increase at the same time, sodium > water (8, 9) (Iatrogenic excessive sodium intake)	No electrolyte water loss (diabetes insipidus)/loss of skin and respiratory tract (8, 9)
Laboratory examination	Urinary osmotic concentration: 300–600 mOsmol/kg, Urinary sodium > 20 mEq/L (7)	Urinary osmotic concentration > 400 mOsmol/kg, Urinary sodium > 20 mEq/L (8, 9)	Central diabetes insipidus: Urinary osmotic concentration < 300 mOsmol/kg, Urine sodium is variable: Skin or respiratory tract: Urinary osmotic concentration >800 mOsmol/kg, Urine sodium is variable (8)
Treatment	Hemodynamic instability: (0.9% NaCl) solution; hemodynamic stability: hypotonic solution (8)	Stop infusion of pathogenic drugs and use diuretics and 5% glucose solution (8)	Rehydration after assessment of water deficit (5% glucose solution) (9): Central diabetes insipidus: Desmopressin (22)

### Rate of correction of hypernatremia

The rate of blood sodium correction in chronic hypernatremia should not be too fast. Sterns (18, 23) recommend that the rate of sodium correction should not exceed 0.5 mEq/L/h, with absolute changes of < 10 mEq/L/day. Fang et al. (24) showed a significantly increased risk of cerebral edema in children with a faster rate of sodium reduction (1.0 vs. 0.5 mEq/L/h). However, another retrospective cohort study involving 250 adult patients with chronic hypernatremia showed that the 30-day mortality rates in the slow correction (24-h correction ≤ 12 mEq/L) group were similar to those in the fast correction (24-h correction > 12 mEq/L) group (25 vs. 28%, *P* = 0.80), and no increased risk of seizures, altered consciousness, or cerebral edema was found to be associated with rapid correction of hypernatremia (25). Data from children suggest that rapidly lowering blood sodium concentrations in chronic hypernatremia has no benefit and may result in seizures, cerebral edema, and irreversible neurological damage (9, 18).

There is no conclusive evidence that rapid correction of acute hypernatremia is harmful. To avoid osmotic demyelination caused by rapidly elevated serum sodium concentrations, the international recommendation for hypernatremia occurring within hours should be to rapidly reduce serum sodium to near normal levels, while for hypernatremia occurring within 1–2 days, the rate of sodium reduction should be 1–2 mEq/L/h (18). Carlberg et al. (26) reported a case of a patient who developed acute hypernatremia (Na^+^ > 180 mEq/L) after consuming a large amount of soy sauce. The patient was treated with 6 L of free water within 30 min and survived without sequelae. However, in a study that explored the effects of overcorrection of acute hypernatremia on patients, a serum sodium correction rate > 1 mEq/L/h (HR = 1.89; 95% CI: 1.03–3.47) was a cause of death in critically ill patients (27). Given the above contradictory results, large-scale prospective randomized controlled trials are needed for further evaluation in the future.

The effectiveness of conventional therapy depends on calculating the loss of electrolytes and water and supplementing the loss. The therapeutic effect in critically ill patients is limited due to the limitations of calculation accuracy and fluid replacement (28). In a retrospective cohort study analyzing 131 patients with moderate hypernatremia (serum sodium ≥ 155 mEq/L) receiving conventional treatment, the 72-h remission rate was only 27% (29).

## Application of CRRT in brain injury patients with hypernatremia

### Efficacy and safety of CRRT in the treatment of hypernatremia

Continuous renal replacement therapy can slowly, controllably, and continuously reduce blood sodium, and it plays an increasingly prominent role in the treatment of acute and severe hypernatremia (11). Park et al. (30) reported a case of hypernatremia complicated with congestive heart failure that gradually improved after continuous veno-venous hemodiafiltration (CVVHDF). Our center (31) administered CRRT treatment to nine burn patients with hypernatremia after conventional treatment was ineffective. Finally, eight patients' serum sodium concentrations returned to normal and they survived. In addition, a retrospective analysis of 95 patients with acute severe hypernatremia in our center showed that CRRT had a better effect on reducing serum sodium than conventional medication and significantly improved the 28-day survival rate of patients (34.8 vs. 8.7%, *P* = 0.002). There were four cases of congestive heart failure in the conventional treatment group, indicating that there was a certain risk for ill patients being given a large amount of fluid replacement (32). These findings confirm the safety and efficacy of CRRT in the treatment of hypernatremia. There is still a lack of prospective randomized controlled trials to evaluate the efficacy and safety of CRRT in the treatment of hypernatremia.

### Indications and timing of CRRT in the treatment of hypernatremia

The Kidney Disease: Improving Global Outcomes (KDIGO) guidelines recommend that patients with acute kidney injury (AKI) complicated by increased intracranial pressure and brain edema caused by acute brain injury should use CRRT instead of conventional dialysis (33). The incidence of AKI in patients with acute brain injury ranges from 8 to 23%, and CRRT should be administered promptly once AKI has occurred (34). Therefore, Yessayan et al. (35) recommended initiating CRRT in patients with brain injury complicated by severe hypernatremia (serum sodium > 165 mEq/L) or renal failure. Park et al. (30) recommended the early use of CRRT to avoid complications such as pulmonary edema caused by conventional fluid replacement therapy. However, the optimal intervention timing of CRRT in the treatment of hypernatremia is still uncertain, and further research is needed.

### The pattern of CRRT

Three patterns of CRRT are often used to provide volume removal and solute clearance, including continuous veno-venous hemofiltration (CVVH), continuous veno-venous hemodialysis (CVVHD), and continuous veno-venous hemodialysis filtration (CVVHDF). CVVHD can effectively remove low molecular weight solutes, and the removal rate decreases with increasing molecular weight. The clearance of solute by CVVH was affected by the pore size of the hemofiltration membrane. The clearance of lower and higher molecular weight solutes was similar until the molecular radius of the solute was close to the pore size of the membrane (11). Blood sodium is a low molecular weight solute that can be effectively removed by all three of the above modes.

Parakininkas and Greenbaum (36) compared the clearance of low molecular weight solutes in pediatric patients using the three CRRT (CVVH, diluted CVVH, and CVVHD) patterns at low blood flow rates. CVVHD was slightly higher than predilution CVVH in the clearance of small molecular solutes but similar in efficiency to diluted CVVH. A meta-analysis included 19 randomized controlled trials (16 of which were studies of continuous RRT treatment) comparing the effects of different CRRT modalities used to clear solutes of different molecular weights and found that hemofiltration increased clearance of moderate to large molecules but did not improve clinical outcomes (37). Currently, there is insufficient evidence to suggest that any one mode of CRRT is superior to other methods for removing low molecular weight solutes, and further evaluation is needed.

### Prescription setting of CRRT in the treatment of hypernatremia

Yessayan et al. (35, 38) suggested adjusting the sodium concentration in the CRRT replacement fluid by adding 23.4% hypertonic saline (containing 4 mEq sodium per mL) to control the patient's serum sodium to the required correction level so that the hypernatremia could be corrected safely and gradually. The above measures changed the dose of CRRT replacement fluid, and serum sodium was monitored every 1–2 h during treatment (31, 34, 35, 39). The concentration of sodium in the replacement solution can be calculated by the following formula:


$$\begin{array}{l} CRRT\left[Na^{+}\right]=\frac{Target\;serum\left[Na^{+}\right]}{\left(1-e^{\left(-Cl\times 24h\right)/V}\right)}+Initial\;serum\left[Na^{+}\right], \end{array}$$

Cl depends on predilution or postdilution:


$$\begin{array}{l} \text{Cl Predilution}=\frac{Qb}{Qb+Qrf}\times SC_{Na_{+}}\times \left(Qrf+Quf\right); \end{array}$$

$$\begin{array}{l} \text{Cl Postdilution}=SC_{Na_{+}}\times \left(Qrf+Quf\right), \end{array}$$

[e is a constant, Cl is the Na+ filter clearance, V is the total body water, the target serum [Na^+^] is negative in the treatment of hypernatremia, Qb is the blood flow rate (L/h), Qrf is the replacement fluid flow rate (L/h), Quf is the ultrafiltration rate (L/h), SCNa is the Na+ sieving coefficient (~1)].

The content of 23.4% hypertonic sodium added to the CRRT replacement solution can be calculated by the following formula:


$$\begin{array}{l} {}[{\text{Na}}^{\text{+}}]\text{content}=\text{Volume of CRRT replacement solution}\times \\ (\text{Target CRRT replacement fluid}[{\text{Na}}^{\text{+}}]–\text{Initial CRRT}\\ \text{ replacement fluid}[{\text{Na}}^{\text{+}}]). \end{array}$$

Paquette et al. (38) successfully treated a patient with acute renal injury with severe hypernatremia by adjusting the sodium concentration of the replacement fluid. Hamdi et al. (40) also treated 11 patients with hypernatremia complicated by acute liver failure and acute renal failure by adjusting the concentration of replacement fluid (blood flow of 60 ml/min and dialysis flow of 400 ml/min). Nine patients survived, with a survival rate of 81.8%.

In addition, our center (31) stated that 3% NaCl solution could be added to the replacement fluid to adjust the sodium concentration of the replacement fluid to reduce the serum sodium concentration (the flow rate of the replacement fluid was 2 L/h, and the blood flow rate was 200 mL/min). The original sodium level in the replacement fluid was set to be lower than the serum sodium level of 8 mmol/L, and then the sodium concentration of the replacement fluid was reduced by 2.47 ± 0.24 mmol/L every 4 h. This scheme was used to treat nine patients with severe burns complicated by severe hypernatremia; eight patients survived, and the survival rate was 88.9%. Adjusting the sodium concentration of the replacement fluid can be calculated by the following formula: $y=480\times \frac{\left(x+0.9\right)}{\left(4+x\right)}+34.5$, where y is the sodium concentration of the replacement fluid (in mEq/L) and x is the added volume of 3% NaCl solution (in L).

### Anticoagulation of CRRT in brain injury patients with hypernatremia

Anticoagulation is a key technology of CRRT. A series of studies have shown that compared with heparin or low molecular weight heparin anticoagulation, sodium citrate anticoagulation can significantly prolong the useful life of the filter and reduce the risk of bleeding (34, 41, 42). KDIGO guidelines recommend that regional sodium citrate should be preferred for anticoagulation in patients with no contraindications to sodium citrate. Patients with brain injury are often accompanied by a high risk of bleeding, and sodium citrate anticoagulation does not affect blood coagulation in the body (33). However, sodium citrate contains a high concentration of sodium and can cause complications such as hypernatremia (43). In a retrospective cohort study at our center (44), the efficacy and safety of anticoagulation with sodium citrate vs. the non-anticoagulant mode of CRRT treatment was evaluated in 64 patients with hypernatremia combined with high-risk bleeding, and no significant difference was found in the efficiency of serum sodium reduction between the two groups. The mean serum sodium concentrations after treatment in the two groups were 151.9 ± 9.8 and 148.4 ± 7.9 mEq/L, respectively; *P* = 0.117, but the incidence of filter failure was significantly lower in the sodium citrate anticoagulated group than in the non-anticoagulated group (2.4 vs. 65.2%). Therefore, sodium citrate anticoagulation can be used as the preferred anticoagulation method for patients with brain injury with hypernatremia treated with CRRT.

## Conclusion

The mortality rate of patients with brain injury complicated by severe hypernatremia is high, and appropriate conventional therapy should be selected according to the etiology and volume state of hypernatremia. The rate of sodium reduction depends on whether it is acute hypernatremia or chronic hypernatremia. CRRT can slowly, safely, and effectively correct hypernatremia and significantly improve the prognosis of patients. Early initiation of CRRT may lead to clinical benefits in patients with brain injury associated with severe hypernatremia (serum sodium > 165 mEq/L) or renal failure. During treatment with CRRT, the changes in blood sodium and the concentration of sodium in the replacement solution should be monitored regularly. Regional sodium citrate anticoagulation therapy can be the preferred choice for patients with brain injury complicated by hypernatremia.

## Author contributions

HW conceived the idea, collected the information, and organized the bibliography. HW, MB, and SS designed of the review article and wrote the text. XL and YX made the final revision. All authors contributed to the article and approved the submitted version.

## Conflict of interest

The authors declare that the research was conducted in the absence of any commercial or financial relationships that could be construed as a potential conflict of interest.

## Publisher's note

All claims expressed in this article are solely those of the authors and do not necessarily represent those of their affiliated organizations, or those of the publisher, the editors and the reviewers. Any product that may be evaluated in this article, or claim that may be made by its manufacturer, is not guaranteed or endorsed by the publisher.
